# Characterization of the *Anopheles gambiae* octopamine receptor and discovery of potential agonists and antagonists using a combined computational-experimental approach

**DOI:** 10.1186/1475-2875-13-434

**Published:** 2014-11-18

**Authors:** Kevin W Kastner, Douglas A Shoue, Guillermina L Estiu, Julia Wolford, Megan F Fuerst, Lowell D Markley, Jesús A Izaguirre, Mary Ann McDowell

**Affiliations:** Department of Computer Science and Engineering, University of Notre Dame, Notre Dame, IN USA; Department of Chemistry and Biochemistry, University of Notre Dame, Notre Dame, IN USA; Department of Biological Sciences, Eck Institute for Global Health, University of Notre Dame, Notre Dame, IN USA

**Keywords:** *Anopheles gambiae*, Octopamine receptor, Homology modelling, Virtual screening, Molecular dynamics, G-protein coupled receptors

## Abstract

**Background:**

Octopamine receptors (OARs) perform key functions in the biological pathways of primarily invertebrates, making this class of G-protein coupled receptors (GPCRs) a potentially good target for insecticides. However, the lack of structural and experimental data for this insect-essential GPCR family has promoted the development of homology models that are good representations of their biological equivalents for *in silico* screening of small molecules.

**Methods:**

Two *Anopheles gambiae* OARs were cloned, analysed and functionally characterized using a heterologous cell reporter system. Four antagonist- and four agonist-binding homology models were generated and virtually screened by docking against compounds obtained from the ZINC database. Resulting compounds from the virtual screen were tested experimentally using an *in vitro* reporter assay and in a mosquito larvicide bioassay.

**Results:**

Six *An. gambiae* OAR/tyramine receptor genes were identified. Phylogenetic analysis revealed that the OAR (AGAP000045) that encodes two open reading frames is an α-adrenergic-like receptor. Both splice variants signal through cAMP and calcium. Mutagenesis analysis revealed that D100 in the TM3 region and S206 and S210 in the TM5 region are important to the activation of the GPCR. Some 2,150 compounds from the virtual screen were structurally analysed and 70 compounds were experimentally tested against AgOAR45B expressed in the GloResponse™CRE-*luc2P* HEK293 reporter cell line, revealing 21 antagonists, 17 weak antagonists, 2 agonists, and 5 weak agonists.

**Conclusion:**

Reported here is the functional characterization of two *An. gambiae* OARs and the discovery of new OAR agonists and antagonists based on virtual screening and molecular dynamics simulations. Four compounds were identified that had activity in a mosquito larva bioassay, three of which are imidazole derivatives. This combined computational and experimental approach is appropriate for the discovery of new and effective insecticides.

**Electronic supplementary material:**

The online version of this article (doi:10.1186/1475-2875-13-434) contains supplementary material, which is available to authorized users.

## Background

Despite decades of research and multiple initiatives, vector-transmitted diseases remain a major public health threat throughout the world. Blood-feeding insects transmit some of the most debilitating infections known to mankind, including malaria, lymphatic filariasis, yellow fever, and leishmaniasis. Malaria is the most deadly vector-borne disease in the world, threatening about 40% of the world’s population and causing nearly one million deaths, primarily in African children [1]. Anopheline mosquitoes are the primary vectors of *Plasmodium* parasites, the causative agents of malarial disease to humans. Although the implementation of artemisinin-based combination therapies in the mid-1990s helped to reduce the global mortality and morbidity due to malaria, vector control has been the cornerstone of malaria control programs, primarily through the use of insecticide-treated bed nets and to a lesser extent, indoor residual spraying. The recent emergence of artemisinin resistance in *Plasmodium falciparum* [2] underscores the role of vector control for reducing the devastation of malaria world-wide. Unfortunately, insecticide resistance is increasing globally, requiring development of more effective insecticides [3].

Octopamine and tyramine are related biogenic monoamines that act as neurohormones, neuromodulators and neurotransmitters in invertebrates [4, 5], performing essential functions and modulating many crucial physiological processes. These processes include learning and memory, locomotion, feeding behaviours, the pheromone response, and cardiac function. In addition, octopamine plays this physiological role in invertebrates only [4, 5]. These reasons make octopamine receptors (OARs) excellent targets for developing new and safer insecticides.

Formamides such as dimethylchlordimeform and amitraz mimic octopamine and provide broad-spectrum insecticidal activity [6]. These insecticides have been used in veterinary practice to control ticks for nearly 40 years, however the quest for novel control compounds that target OARs has been relatively ineffective. However, recent technological developments in screening protocols, however, may reverse this trend [4].

OARs are G-protein coupled receptors (GPCRs), a family of seven transmembrane (TM) receptors that are involved in many diseases and are the target of approximately 30% of all modern medicinal drugs [7, 8]. GPCRs are called such because they interact with G-protein trimeric complexes, the three main types being G_αs_, G_αi_, and G_αq_.

Few GPCR crystal structures have been elucidated due to the complexity of these proteins as well as their location in lipid membranes. While the limited crystal structures that have been solved generally have low sequence identity (for example, the sequence identity of the β_2_-adrenergic receptor and rhodopsin is below 20%), the 3D structures of their TM regions are found to be very similar. Large structural differences are generally found in the loop regions, where location and secondary structure between the receptors can deviate [9]. Realizing this characteristic, it is hypothesized that 3D molecular modelling using an existing GPCR model as a template, and performing a simulation on the resulting model, will fix major variations from the existing model and prepare a novel GPCR for virtual screening.

GPCRs are activated via agonists docking to the interior of the receptor near the extracellular side. To date, three residues have been found to be important for the activation of most GPCRs. These are an aspartic acid in the third TM region (TM3) and two of three closely grouped serine residues found in TM5. The endogenous biogenic amine agonist appears to hydrogen bond via its amine group and its hydroxyl groups to the aspartic acid and serines of the GPCR, respectively [10–12].

Studies have been conducted studying OARs of other insects, including the honey bee [13], silkworm [14], cockroach [15], and fruit fly [16, 17]. These have mostly been molecular and functional characterizations of the receptor, though many studies have yielded the effects of common GPCR agonists and antagonists on their respective OARs. Here, an OAR from *Anopheles gambiae* mosquitoes was characterized and novel agonists and antagonists were discovered through molecular dynamics (MD) simulations and virtual screening, followed by larval bioassays with candidate compounds.

## Methods

### Insects and materials

*Anopheles gambiae* (strain PEST) mosquitoes were raised and maintained in an environmental chamber at 26°C, 85% relative humidity, with a 16-hour light, eight-hour dark cycle including a one-hour dusk/dawn period [18]. Larvae were fed daily a 2:1 mixture of fish pellets: brewer’s yeast, that had been finely ground [19]. DL-octopamine, tyramine, dopamine, naphazoline, clonidine, serotonin, chlorpromazine, cyproheptadine, promethazine, all hydrochloride salts, and tolazoline a benzylimidazoline salt, were obtained from Sigma-Aldrich. Metoclopramide hydrochloride was obtained from MP Biomedical. Compounds identified in the virtual screen were purchased from Princeton BioMedical, ChemDiv, Chembridge and Enamine and tested *in vitro* against AgOAR45B expressed in the GloResponse™CRE-*luc2P* HEK293 reporter cell line and in larval bioassays.

### Expression analysis of *AgOAR45A*and *AgOAR45B*

Total RNA was isolated from five different *An. gambiae* immature stages (L1-P), adult females and males, adult female heads only, and adult female abdomen/thorax using the RNeasy Mini Kit (Qiagen). The DNase (Fermentas)-treated RNA was used to generate cDNA using Superscript III (Invitrogen) and oligo (dT_12–20_), according the manufacturer’s recommendations. Quantitative PCR (qPCR) was performed using SYBRGreen (ABI), an ABI 7900 RT-PCR system and 200 ng of cDNA per sample, a final concentration of 0.15M of each primer, and an annealing temperature of 60°C. Primer sets used for expression analysis were: Ag10592 forward- CACCATCGAACACAAAGTTGACACTT; Ag10592 reverse- CGAACGTAACGTCACGGCCA; Ag45A&B forward- GGGTACGTCGTCTACTCAGCCCTC; Ag45A reverse- TGTATCCGCAGCGTTAGCCGATTG; Ag45B reverse- CGAGATTGTTCTTGCCACCTTTGGTG. The 40S Ribosomal protein subunit 7 (AGAP01592) was used as an internal control. Reactions for each gene and for the control used were carried out in triplicate. Relative expression levels of each gene was determined by the ΔΔC_T_ method, where relative expression is expressed as a fold difference relative to whole females and expressed as 2^- ΔΔCT^. The following formula was used: ΔΔC_T_ = ΔC_T(stage or condition)_ − ΔC_T(Females)_ and ΔC_T_ = C_T (gene of interest)_ − C_T (40S RNA)_.

### Heterologous expression of AgOAR45B octopamine receptor

Total RNA was isolated from heads of three-day old adult females using RNeasy Mini Kit (Qiagen). cDNA was synthesized using SuperScript III (Invitrogen), and used as a template for PCR amplification of the *AgOAR45A* and *AgOAR45B* genes. Insertion of the coding sequences into the *Sgf*I and *Pme*I sites of the pF9a CMV hRluc-neo vector (Promega) was performed by digestion of a fragment amplified by the following primers: Ag45forward- TAAAGCGATCGCCATGAACGAGTCGGAGTGTGCC; Ag45Areverse- TTGTGTTTAAACTCTCGAGTCGGACAGGTCGC; Ag45Breverse- CGCGGTTTAAACTCTGAACACACCACCGACGA. Primers were constructed based on the annotated sequence of the AGAP000045 gene (VectorBase, Protein ID: AGAP000045-PA and -PB [20].

GloResponse™CRE-*luc2P* HEK293 reporter cell line (Promega) were maintained as adherent culture at 37°C, 5% CO_2_ in DMEM (Invitrogen) supplemented with 10% fetal calf serum (Atlanta), and 50 mg/ml hygromycin B. Transfection of cells was carried out using the Amaxa Nucleofector kit per the manufacturer’s instructions. Control transfections were performed using a pF9A plasmid with the barnase (Bacterial Ribonuclease) gene removed as suggested by the manufacturer (Promega). Stable lines were created by applying 400 mg/ml G418 for three weeks. Stable clones of AgOAR45B expressing HEK293 reporter cells were created through two rounds of limiting dilution cloning.

### cAMP assay

Intracellular cAMP increase was monitored through a CRE-luc reporter construct in HEK293 cells (Promega). Stable cell lines were plated at a density of 4×10^4^ cells per well in white 96-well assay plates (Corning, Cat. #3917). Cells were immediately treated with the various compounds, and returned to the incubator for four hours before being assayed. cAMP was quantified through luciferase production using the Dual-Glo luciferase assay kit (Promega) following the manufacturer’s instructions. Luciferase units were normalized to the cell number using the internal Rluc construct in the pF9A expression plasmid.

### Intracellular Ca^++^ assay

Stable cell lines expressing *AgOAR45A* or *AgOAR45B* were plated in black well, clear bottom, 96-well assay plates (Greiner Bio-One, Cat. #655090) at 4×10^4^ cells per well. Cells were allowed to grow overnight before being assayed. Cells were preloaded with Fluo-4 NW (Molecular Devices), and carried out per the manufacturer’s instructions. Fluorescence was monitored before and after addition of compounds using the Flexstation3 (Molecular Devices), at two-second intervals for 120 seconds.

### Site-directed mutagenesis of *AgOAR45B*

The *AgOAR45B* gene was subcloned into a TA vector (Invitrogen), according to the manufacturer’s instructions. Site-directed mutagenesis of *AgOAR45B* was carried out using Quick Change Lightning Kit (Agilent). Primers (IDT) were designed to introduce single amino acid changes (see Additional file 1). Double mutants were created through two rounds of mutagenesis. Mutations were confirmed through automated sequencing (ND Genomics Core Facility). Mutant genes were then excised and moved to the pF9A expression vector as described above.

### Membrane preparation and radioligand binding assay

Cells in T75 flasks were washed with 10 ml of PBS, removed by scraping, centrifuged at 500 g, and then resuspended in 5 ml of lysis buffer, 50 mM Tris-Cl pH 7.4. Cells were incubated on ice for 10 min and homogenized with a Dounce homogenizer with 30 strokes. The homogenate and 5 ml wash with lysis buffer were then centrifuged at 23,000 g for 30 min at 4°C to pellet crude membranes. The resulting pellet was re-suspended in 50 mM Tris-Cl pH 7.4, 5 mM MgCl2, 0.5 mM EDTA pH 7.4 and quantified by micro BCA assay (Thermo).

Radioligand binding assays were carried out using ^3^H-Yohimbine to determine binding affinity of the octopamine receptor with different compounds. Isolated membranes, 30 μg per well, were incubated in the presence of 16 nM ^3^H-Yohimbine (American Radiolabeled Chemicals) in binding buffer, and various concentrations of compounds. The final reaction volume was 125 ml per well in a 96-well assay plate. One-hundred mM clonidine was used to determine non-specific binding in each experiment. After a two-hour incubation, membranes were collected and washed on filter mats, pretreated with 0.3% polyethylenimine (Sigma) using a Brandel 96-well harvester and counted in a Perkin Elmer Microbeta counter.

### Larval bioassay

Dose response curves were made to determine the LD_50_ for some compounds against three-day old *Aedes aegypti* larvae. Ten larvae were put in each well of a 12-well plate, with each well containing a different concentration of compound in water. Three replicate wells were made for each experiment and the curves performed three separate times. Control wells were included in each experiment, which contained only DMSO in water. Plates were incubated 24 hours in standard insectary conditions. Mortality was determined after 24 hours.

### Project workflow

A computational-experimental workflow (see Additional file 2), similar to the computational approach described by Yarnitzky *et al.* [21] was utilized. The method was as follows: 1) homology models, both inactive (antagonist-based) and active (agonist-based) conformations, were created using the fragment-based method I-TASSER (Iterative Threading Assembly Refinement) [22–24] as well as surrounded by lipids, water, and ions using visual molecular dynamics (VMD) [25]; 2) a preliminary virtual screen with one of two compound test sets (composed of either GPCR agonists or antagonists whose activity on AgOAR45B was experimentally determined using an AgOAR45B reporter assay) was performed and a set of top-ranked positions of the most were chosen and used in MD simulations with the proper active (agonist-binding) and inactive (antagonist-binding) protein conformations; 3) MD simulations were performed until stable ligand positions were obtained, then an additional virtual screen with the test set was performed on each resulting protein conformation and the results were analysed manually. Step 3 was repeated until the ligand positions were stabilized in the protein even after ten additional nanoseconds (ns) of simulation; 4) final conformations were then used to build grids which were subjected to virtual screening using the ZINC library; and, 5) the resulting compounds were analysed and compounds with differing structural characteristics were purchased and experimentally tested *in vitro*.

### Homology modelling

Both initial inactive (antagonist-based) and active (agonist-based) conformations of AgOAR45B were generated using the I-TASSER online server [26]. For the inactive conformation, a structure was obtained that was built based on many GPCR antagonist-bound and inverse agonist-bound conformations, primarily a crystal structure of β_2_-adrenergic receptor with partial inverse agonist carazolol bound [PDB:2RH1]. For the active conformation, a structure was obtained that was built based on the crystal structure of the β_2_-adrenergic receptor-G_αs_ protein complex with high affinity agonist BI-167107 bound [PDB:3SN6]. Both conformations were obtained using standard I-TASSER settings. In the case of the active conformation of AgOAR45B, 3SN6 (chain R) was used as a template to generate the model. Root-mean-square deviations (RMSDs), as a measure for protein stability, were calculated using the VMD 1.9 RMSD Trajectory Tool [25]. AgOAR45B has a 24% sequence identity (35% similarity) to the β_2_-adrenergic receptor (see Additional file 3). The active site residues were determined from analyzing the inactive and active β_2_-adrenergic receptor’s crystal structures and observing residues that interacted or had the potential to interact with the crystal structures’ bound ligands [27, 28]. The active site sequence identity is much higher at 67% (73% similarity).

### Molecular dynamics

Using VMD 1.9, each virtual representation of the protein was first embedded in a large 1-palmitoyl-2-oleoylphosphatidylcholine (POPC) lipid bilayer, removing any lipid that overlapped with the protein. The virtual protein-membrane system was then solvated with TIP3P water molecules, and neutralized by virtually adding KCl up to 400 mM. Initially, CHARMM 27 parameters [29] were assigned to all molecules using VMD 1.9 to enable the addition of the lipid bilayer, water and ions. However, once each virtual system (both active and inactive conformation) was prepared and its respective ligand (octopamine for the active conformation and promethazine for the inactive conformation) was ready to be added, AMBER gaff and ff03.r1 parameters were assigned to the ligand and the rest of the molecules, respectively, using Amber 11 tleap [30–32]. The AMBER force field was chosen as it allows the generation of parameters for the ligand using the antechamber module [33]. A disulphide bridge was added between the residues of Cys93 and Cys194, as this bridge also existed in the template PDBs.

Each complete virtual system consisted of the respective conformation of AgOAR45B embedded to a large POPC bilayer with 168 lipid molecules. In each virtual system, all residues were at the normal protonation state for physiological pH. In addition, the antagonist- bound system contained 171 potassium ions, 204 chloride ions, and 22,525 water molecules for a total of 99,706 atoms (measured 95×96×128 Å), while the agonist-bound system contained 172 potassium ions, 205 chloride ions, and 22,654 water molecules for a total of 100,077 atoms (measured 101×92×127 Å). Before MD simulations the systems were equilibrated using 120 CPU cores as follows: 1) MD of lipid tails for 500 picoseconds (ps) [time step = 2 femtoseconds (fs)] with protein, ligand, lipid head groups, water, and ions kept fixed; 2) equilibration for lipids, water and ions for 500 ps (time step = 2fs) with harmonic constraints on the protein and ligand; 3) equilibration of the entire system for 500 ps (time step = 2fs) with no molecular constraints. After equilibration, 20–30 ns of MD simulation were performed using 504 CPU cores in two to three 10 ns increments, with time step = 2fs and trajectory data being collected every 200 ps. The equilibration and simulation steps were run using NAMD 2.8 [34] on the high-performance computing cluster Kraken [35].

### Virtual screen preparation

All virtual screening jobs were run on their respective ‘protein only’ homology models (i.e., containing no lipids, waters or ions). The proteins were prepared by first running the Protein Preparation Wizard workflow [36, 37], and then grids were generated using Glide’s Receptor Grid Generation application, each found in Schrodinger Suite 2011’s Maestro [38]. To obtain initial ligand poses used in grid generation, each of the conformations were first overlapped in PyMOL [39] with the top templates used by I-TASSER for their creation (2RH1 for the inactive conformation and 3SN6 for the active conformation) and the positions of the ligands found in each of the templates were first saved as a PDB file, then added using Schrodinger Suite 2011’s Maestro to their respective AgOAR45B conformation (2RH1 partial inverse agonist CAU was used for the inactive conformation and 3SN6 agonist 30G was used for the active conformation) to denote the active site of the protein. The ligand added to each of the homology models was used as the centroid of the grid, determining the area in which the libraries of compounds should be docked. No constraints were used.

### Virtual screening with known GPCR ligands

The inactive and active conformations of AgOAR45B with the added ligands were used to build the grids, which were then run in a virtual screen using Schrodinger’s Glide software [40–43] against known GPCR antagonists and agonists, respectively. The original compound structures used in the two test sets were downloaded from the NIH’s PubChem website. One of the test sets contains known GPCR agonists: synephrine, cinnamic acid, clonidine, demethylchlordimeform, dopamine, eugenol, histamine, naphazoline, norepinephrine, octopamine, phentolamine, serotonin, tolazoline, transanethole, and tyramine. The other containing known GPCR antagonists: rauwolscine, demethylchlodimeform, 8-hydroxymianserin, amitriptyline, antazoline, chlorpromazine, cyproheptadine, desipramine, desmethylmianserin, dihydroergotamine, gramine, imipramine, maroxepin, metoclopramide, mianserin, phentolamine, prazosin, promethazine, propranolol, triprolidine, and yohimbine. Each of the test sets was then prepared using Schrodinger Maestro’s LigPrep [44] to generate different potential protonation states at pH range 7–8, tautomers and ring conformations. After each of the test sets was docked to its respective AgOAR45B conformation, top pose positions for the antagonist promethazine and the agonist octopamine were saved and later added to the inactive (antagonist-bound) and active (agonist-bound) conformations, respectively. These protein conformations with docked ligands were then used as the starting position for the MD simulations.

### Virtual screening with ZINC library

The library used in the docking contained drug-like compounds from the ZINC online database [45]. Compounds were prepared using Schrodinger Maestro’s LigPrep to generate different potential protonation states at pH range 5–9, tautomers and ring conformations. The final library contained approximately 12 million compounds. It was split into five sublibraries (∼2.4 million compounds per sublibrary) for the first run of high-throughput virtual screening.

The library was screened against five grids of AgOAR45B (two from the antagonist-bound conformations and three from the agonist-bound conformations), built from the receptor positions after 20 ns of MD simulation each. Virtual screening using the ZINC library was performed using Schrodinger’s Glide software in three steps: 1) each of the five sublibraries was filtered using high-throughput virtual screening and the top 30,000 compounds in each sublibrary were saved and recombined making a library containing 150,000 compounds; 2) this new library was then filtered further using standard precision virtual screening and the top 15,000 compounds were saved; and, 3) this new library was then filtered one more time using extra precision virtual screening and the top 1,500 compounds were saved and analysed, considering the score, the relevant interactions in the active site pocket and diversity in the sampling for further testing.

## Results

### Molecular characterization of *Anopheles gambiae*octopamine receptors

*Anopheles gambiae* OAR and the closely related tyramine receptor (TyrR) genes were identified by homology searching utilizing an OAR sequence (*DmOamb*) from *Drosophila melanogaster*. Seven possible *An. gambiae* OAR/TyrR genes were identified in VectorBase, AGAP000045, AGAP002519, AGAP002888, AGAP002886, AGAP004034, AGAP013324, and AGAP011698. One locus, AGAP011698, contained only approximately one-half of the predicted coding sequence of *DmOamb* and, although likely encoding an OAR or TyrR, was eliminated from further analysis. The closest homologue to *DmOamb* was AGAP000045. Similar to the *DmOamb* locus that encodes for two alternatively spliced genes, *DmOamb-D* and *DmOamb-B*, the AGAP000045 locus encodes two open reading frames (ORF), AGAP000045_OARA (*AgOAR45A)* of 1,779 bp and AGAP000045_OARB (*AgOAR45B)* of 1,773 bp, that encode putative proteins consisting of 592 and 590 amino acids, respectively. The splice variants have a deduced amino acid identity of 56.1%, with the first three exons being identical and the fourth and largest (1,046 bp for *AgOAR45A* and 1,040 bp for *AgOAR45B*) exons being alternatively spliced (see Additional file 4A and B). The hydropathy plot of both AgOAR45A and AgOAR45B revealed seven hydrophobic domains, indicative of seven transmembrane-spanning regions typical of GPCRs (see Additional file 4C). In addition, both predicted proteins contain the GPCR characteristic aspartic acid (D) in TM3 and two serine (S) residues in TM5.

### Phylogenetic analysis

**Figure 1:**
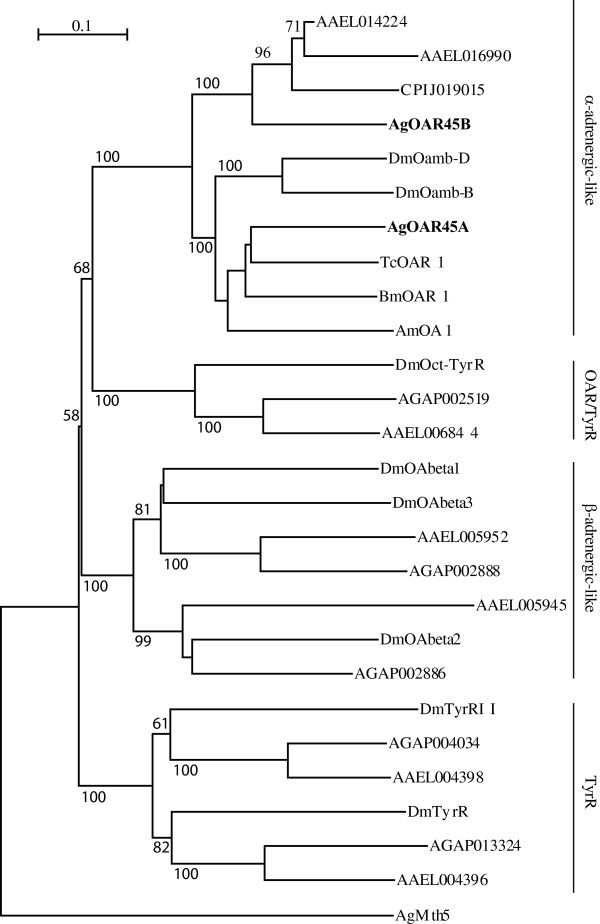
**Phylogenetic relationship of** ***Anopheles gambiae*** **AgOAR45A and AgOAR45B with other octopamine (OAR) and tyramine (TyrR) insect receptors.** Sequences from *An. gambiae* (AGAP000045, AGAP002519, AGAP002888, AGAP002886, AGAP004034, AGAP013324; *Ae. aegypti* (AAEL004398, AAEL004396, AAEL006844, AAEL014224, AAEL016990, AAEL005952, AAEL005945), and *Cu. quinquefasciatus* (CPIJ019015) were downloaded from VectorBase. Sequences from *D. melanogaster* [DmTyrRII (CG16766), DmTyrR (CG7431), DmOct-TyrR (CG7485), DmOamb-B (CG3856-RB), DmOamb-D (CG3856-RD), DmOAbeta1 (CG6919), DmOAbeta3 (CG42244) and DmOAbeta2 (CG33976)] were downloaded from FlyBase. Additional sequences, from *Bombyx mori* [BmOA1 (NP_001091748.1)], *Apis mellifera* [AmOA1 (NP_001011565.1)], and *Tribolium* [TcOAR1 (DAA64496.1)] were downloaded from GenBank. The alignment was performed on complete amino acid sequences and calculated using the ClustalX 2.1 [49]. The tree was constructed using ClustalX 2.1, and the neighbor-joining algorithm with a bootstrap value of 1,000. Numbers on branches are the percentage of bootstrap support for each branch node, only those above 50% are represented on the trees. The scale represents the rate of amino acid substitution per site. AgOAR45A and Ag OAR45B are in bold.

A phylogenetic tree was constructed using the ClustalX 2.1 and the neighbour joining method of 21 OARs and TyrRs from *An. gambiae*, *Ae. aegypti*, *Bombyx mori*, *Culex quinquefasciatus,* and *D. melanogaster*. The AGAP000045 predicted proteins group with the α-adrenergic-like receptors, such as *DmOamb* (Figure 1), exhibiting a 34.9-37.5% deduced amino acid identity similarity with the *DmOamb* splice variants, a 20.7-21% similarity to *DmOct-TyrR,* a 10.8-18.2% similarity with the *Drosophila* β-adrenergic-like receptors, and a 19.5-21.7% similarity to the *Drosophila* TyrR. AGAP002886 and AGAP002888 likely encode for β-adrenergic-like receptors, AGAP002519 an OAR/TyrR and AGAP004034 and AGAO013324 each a TyrR (Figure 1).

### Expression of *AgOAR45B*

**Figure 2:**
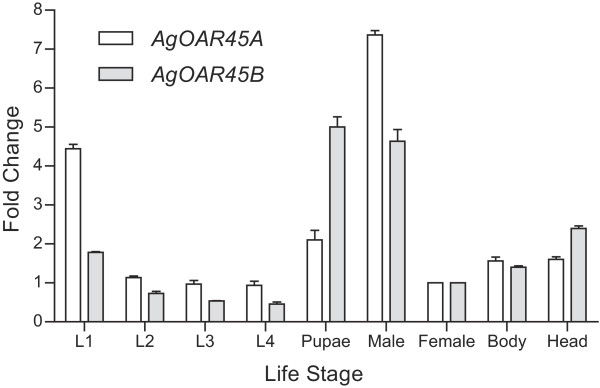
**Expression levels of** ***AgOAR45A*** **and** ***AgOAR45B.*** Quantitative PCR analysis of *An. gambiae AgOAR45A* (grey) and *AgOAR45B* (white) at different life stages, including larvae (L1-L4), pupae, whole body adult females and males, adult female heads and thorax/abdomen. Data are expressed as fold change relative to whole females. Means ± SD of three independent experiments are presented.

Quantitative PCR was performed to assess the expression pattern of *AgOAR45A* and *AgOAR45B* at different developmental stages and tissues of *An. gambiae* mosquitoes. Both genes were expressed in all life stages, with the lowest expression being in the larval stages. Importantly, both *AgOAR45A* and *AgOAR45B* were expressed in female heads (Figure 2).

### Functional characterization of AgOAR45A and AgOAR45B

To functionally characterize AgOAR45A and AgOAR45B, cDNAs of both proteins were separately cloned and stably expressed into the GloResponse™CRE-*luc2P* HEK293 reporter cell line. Compared to the reported sequence in VectorBase, the cloned *AgOAR45A* gene contained five silent mutations and a 6-bp deletion resulting in two fewer glutamines (Q426 and Q427) in a glutamine repeat region. The cloned *AgOAR45B* gene differed from the VectorBase sequence with two silent single nucleotide polymorphisms (SNPs), two SNPs resulting in a valine (V) at position 45 instead of an alanine (A) and a switch from an arginine (R) at position 403 to a glutamine (Q), a 3-bp deletion resulting in the lack of one histidine in a histidine repeat region and an insertion of a valine (V) at the end of the sequence, due to the cloning design.

**Figure 3:**
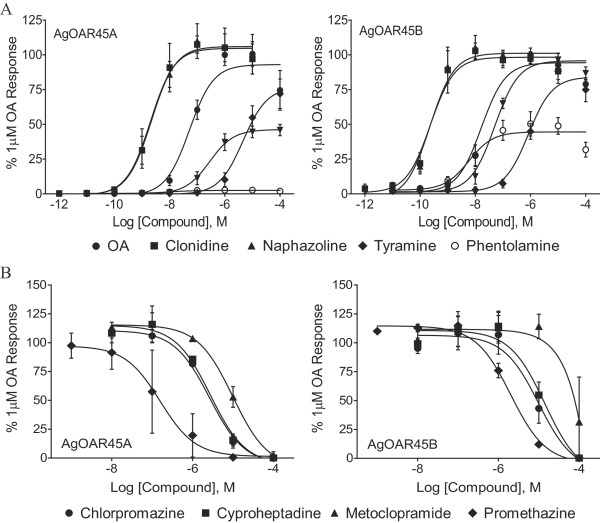
**Pharmacological characterization of** ***Anopheles gambaie*** **octopamine receptors AgOAR45A and AgOAR45B in HEK293-CRE-Luc cells. A**. Dose response curves of AgOAR45A and AgOAR45B to known octopamine receptor agonists [octopamine (OA; closed circles), clonidine (closed boxes), naphazoline (closed triangles), tyramine (closed diamonds), phentolamine (open circles)]. **B**. Dose response curves of known octopamine receptor antagonists [chlorpromazine (closed circles), cyproheptadine (closed boxes), metaclopramide (closed triangles), promethazine (closed diamonds) of AgOAR45A and in the presence of 1 μM octopamine. Data are expressed as % 1 μM octopamine response. Means ± SD of three independent experiments are presented.

Both genes signaled through cAMP (G_αs_) and exhibited typical OAR pharmacology [4], responding to octopamine and other known octopamine receptor agonists (clonidine, naphazoline, and phentolamine) (Figure 3A) in the nM range and not to other biogenic amines (dopamine and serotonin) (Table 1). As is typical for OARs, both genes exhibited a dampened response to tyramine (Figure 3A). The best agonists were clonidine and naphazoline, inducing a response in the pM range (Table 1), consistent with both receptors being α_2_-adrenergic receptor homologues [4]. The receptor responses to octopamine were also inhibited by synthetic antagonists of OARs (chlorpromazine, cyproheptadine, metaclopramide, and promethazine) (Figure 3B and Table 1), further indicating that both AgOAR45A and AgOAR45B are functional OARs [4].

**Table 1:** **EC/IC**_**50**_ **of chemistries for AgOAR45A and AgOAR45B**

Receptor	AgOAR45A	AgOAR45B
**Agonist**	**EC**_**50**_ **(nM)**	
Octopamine	53	17
Clonidine	2	0.24
Naphazoline	2.2	0.23
Tolazoline	280	5
Tyramine	4600	840
Phentolamine	10	5.8
Serotonin	>50,000 (inactive)	>50,000 (inactive)
Dopamine	>50,000 (inactive)	>50,000 (inactive)
**Antagonist**	**IC**_**50**_ **(μM)**	
Chlorpromazine	2.6	9.9
Cyproheptadine	2.8	14
Metaclopramide	9.8	>50 (inactive)
Promethazine	.16	2.0

To examine the ability of AgOAR45A and AgOAR45B to signal through G_αi_, cAMP activity was assessed in the presence of forskolin in response to octopamine and naphazoline (agonists of OARs) and inhibitory activity was not detected for either receptor. To monitor Ca^2+^ signals (G_αq_ signaling), HEK293 reporter cells stably expressing either *AgOAR45A* or *AgOAR45B* were preloaded with the calcium sensitive dye Fluo-4 before stimulation with octopamine. Both receptors also signal through G_αq_ (see Additional file 5).

### Discovery of five potential binding modes of AgOAR45B

**Figure 4:**
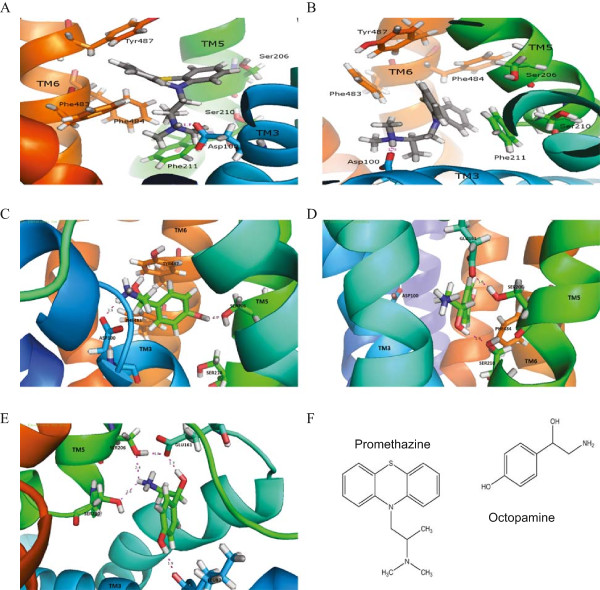
**Protein conformations resulting from molecular docking of active compounds.** The antagonist promethazine can bind to D100 in two different conformations. Promethazine exhibits H-bonding to D100 of TM3 and does not interact with either of the serines of TM5 **(A)** and has potential π -π interactions with either F483 of TM6 or F211 of TM5 **(B)**. The agonist octopamine can potentially H-bond to D100 and S206 **(C)**, S210 **(D)**, or S206, S210, and E161 **(E)**. Octopamine also exhibits potential π -π interactions with either F483 **(C)** or F484 **(D)**. Note that an H-bond between octopamine and L97 is also seen in panel **E**, but was ignored as this is a peptide bond and can thus be made with any amino acid. **(F)** Chemical structures of octopamine and promethazine.

As no functional differences between AgOAR45A and A5OAR45B were detected, *in silico* characterization and screening was performed with only AgOAR45B. Homology models of AgOAR45B were generated for both the inactive (antagonist-bound) and active (agonist-bound) conformations. As expected, the largest differences between the inactive and active conformations were the intracellular and extracellular loops as well as both C and N-termini, including the beginning of the TM1 region. In general, differences in the TM regions of the two conformations were relatively small (see Additional file 6). The models were refined by MD simulations with the most active agonist (octopamine) and antagonist (promethazine) of the test set for the active and inactive conformations, respectively. For each of the four starting positions, MD was run using two different seeds, resulting in eight simulations total. For the inactive conformation, promethazine bound in two different conformations to D100 (Figure 4A and B). Similarly, three different binding modes were found for octopamine after 20 ns MD simulation (Figure 4C-E). Three very different ligand positions were obtained from the simulation of the active conformation, with octopamine binding to either both D100 and S206 (Figure 4C), S210 only (Figure 4D), or simultaneously binding to S206, S210 and E161 (Figure 4E).

Each of the antagonist and agonist-bound homology models were simulated for 20 ns in a combination membrane and water environment that approximately reproduces the protein’s biological surroundings. The simulations were stopped every 10 ns and the stability of the ligands studied. After 20 ns, two of the antagonist-bound active site conformations and three of the agonist-bound active site conformations stabilized. The other simulations were continued for another 10 ns, but no new stable conformations were observed. The stabilization of the active sites of the five generated AgOAR45B conformations was further confirmed via calculation of the RMSDs of the MD simulation trajectories compared to their initial conformations (see Additional file 7). The ligand and active site for each conformation were found to have a very stable interaction for at least ten full nanoseconds (10–20 ns maximum RMSD fluctuation <1 Å).

### Mutagenesis of *AgOAR45B*

**Figure 5:**
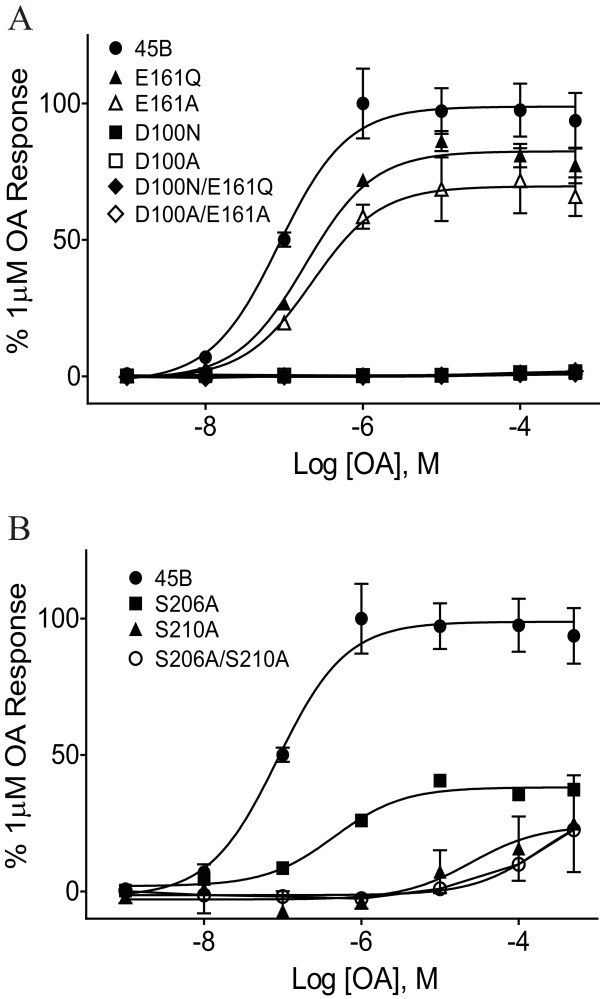
**Pharmacological characterization of AgOAR45B mutants. A**. Dose response curves of optopamine E161Q (closed triangles), E161A (open triangles), D100N (closed squares), D100A (open squares), D100N/E161Q (closed diamonds), and D100A/E161A (open diamonds) double mutants. **B**. Dose response curves of optopamine S206A (closed boxes), S210A (closed triangles) and S206A/S210A double mutants (open circles). The same control wild-type AgOAR45B (closed circles) data is presented in both **A** and **B**. Data are expressed as % 1 μM octopamine response. Means ± SD of three independent experiments are presented.

To identify the receptor amino acid residues important for activity, we performed site-directed mutagenesis analysis of the residues indicated by the homology modelling to be important for binding. A number of studies have identified important residues vital for GPCR agonist binding activity and have indicated the importance of an aspartic acid in TM3 (D100) and serine residues in TM5 (S206 and S210) [10–12] and this has been shown to be true for α-adrenergic-like octopamine receptors [46]. MD simulations of AgOAR45B suggested that a glutamic acid (E161) residue in TM4 may also play a role in ligand recognition. Therefore, AgOAR45B mutants were constructed changing each of these residues to alanine (A) residues by site-directed mutagenesis. The role of the aspartic acid (D100) and the glutamic acid (E161) by mutating these residues into their non-charged amino acids (asparagine (N) and glutamine (Q), respectively) was further assessed. Mutated forms of *AgOAR45B* were generated via site-directed mutagenesis and transiently expressed into the GloResponse™CRE-*luc2P* HECK293 reporter cell line. Dose response curves for octopamine were generated for each single and double mutant receptor (Figure 5). Mutagenesis of either the aspartic acid or the glutamic acid resulted in loss of AgOAR45B to responsiveness to octopamine (Figure 5A). Single mutant analyses revealed that the aspartic acid residue is essential for octopamine activity as mutagenesis of this residue to either an alanine or an asparagine completely abolished octopamine reactivity. The glutamic acid residue, however, is marginally important for the response (Figure 5A). The results also indicate that while both serine residues participate in the octopamine response, S210 is more important than S206 (Figure 5B).

### Discovery of four antagonist-binding scaffolds

Two different antagonist conformations of AgOAR45B were screened against the ZINC library. Of the resulting 1,500 compounds from each of the two conformations, the top 700 compounds from each were analysed and separated these compounds into four primary binding-scaffold groupings which were determined based on the hydrogen bonding of the D100 residue found in the active site of the protein with one of four binding sites in each antagonist: protonated piperazine, protonated imidazole, protonated -NR_2_, and a fourth group with other potential binding scaffolds (see Additional file 8).

### Discovery of four agonist-binding scaffolds

Similarly, three different conformations were used to model the agonist conformation. Of the resulting 1,500 compounds from each of the three conformations, the top 250 compounds from each were analysed and separated these compounds into four binding-scaffold groupings. These scaffolds were determined based on the hydrogen bonding of the D100 residue found in the active site of the protein with one of four binding sites in each agonist: protonated piperazine, protonated -NR_2_/-NR_3_, -NH-R-NH- (where D100 interacts with both nitrogens), and a fourth group with other potential binding scaffolds (see Additional file 9). The other active portion of the compounds appeared to either hydrogen bond with one or the other of the serine residues in the TM5 region, or potentially did π stacking with some of the benzene ring-containing residues in the TM6 region. Both of these residues have been shown to move in the presence of agonists [27, 28] and were thus both considered. Scaffolds where the agonist hydrogen bonded only to the E161 residue were not considered due to E161’s low importance in the activation of the protein as determined from the mutagenesis assays (Figure 5).

### New potential insecticides

From the 2,150 compounds assessed from the two antagonist-bound and three agonist-bound conformations, a total of 70 compounds were chosen and purchased. These compounds were tested *in vitro* against AgOAR45B expressed in the GloResponse™CRE-*luc2P* HEK293 reporter cell line to determine their efficacy in being antagonists or agonists. Ultimately, 21 antagonists, 17 weak antagonists, two agonists, and five weak agonists were identified, while 25 of the 70 compounds were not active in the reporter assay (see Additional files 10 and 11).

Eight of the compounds identified from the antagonist conformation ZINC screen (46140767, 46140913, 12790591, 15671138, 12434335, 4148879, 24757679, and 42437778) inhibited the 1 μM octopamine response of the AgOAR45B receptor reporter assay by greater than 40%, however, these compounds decreased the viability of the HEK293 reporter cells (see Additional files 10 and 11, superscript c).

Additional chemistries that resulted in a reduction in receptor stimulation to 1μM octopamine less than two standard deviations (<80% activity) were chosen for further characterization. Four compounds (65552607, 32860047, 27417161, and 9274026) exhibited a dose response for at least two concentrations. Dose–response curves were generated for 9274026 and 9274026-like compounds (6791891, 9273955, 15725975, and 9274053) using the AgOAR45B receptor reporter assay. As the high concentrations of the 9274026-like compounds were insoluble, IC_50_ values could not be accurately assessed, however, the results indicate that all four of the 9274026-like compounds were able to inhibit the response of AgOAR45B to 1 μM octopamine as well as the 9274026 compound (11% of the 1 μM octopamine response; IC_50_ 13.2 μM; see Additional files 10 and 11). Similarly, five compounds similar to 27417161 (12535485, 32843433, 12484738, 00200688, and 02721570) were selected and dose–response curves were generated. Two of the compounds, 00200688 and 12484738, induced responses similar to 27417161 (~40% inhibition; see Additional files 10 and 11).

Four compounds identified from the agonist-bound confirmation behaved as weak agonists: 00441586-13%, 48194443-3%, 48345912–8.3%, and 33313914–4.2% of octopamine response (see Additional files 10 and 11). Five compounds similar to 48345912 (48354000, 48353672, 4853678, 40118665, and 12984232) were selected for further testing. One of the compounds, 48353678, had agonistic activity higher than to 48345912 (93.4% of octopamine response). These compounds were further assessed for antagonistic activity in the presence of 1μM octopamine and four compounds, 4834512, 48354000, 48353672, and 40118665, were better antagonists than agonists. Compound 48353678, however, exhibited no antagonistic activity (see Additional files 10 and 11).

Two additional chemistries identified in the original *in silico* screens (10883478 and 02643656) were selected. One compound, 02643656, exhibited agonist activity slightly less than 48353678. Compound 10883478 behaved as an antagonist, inhibiting the response of AgOAR45B to 1 μM octopamine by 83%. The 10883478 scaffold was further explored by selecting ten compounds (61715350, 58171610, 58007135, 61715380, 7205872, 11207230, 12547680, 39985747, 12794746, and 32322962) from the ZINC library that were similar to 10883478. Only one (12547680) had antagonistic activity similar to the parent compound. None of the compounds exhibited any agonistic activity (see Additional files 10 and 11).

To assess agonist and antagonist binding to AgOAR45B the best two agonists (48353678 and 02643656) and three antagonists (10883478, 40118665, and 48345912) were selected and competition binding assays with ^3^H-Yohimbine were performed. All the compounds exhibited affinity to AgOAR45B that was similar to octopamine (i.e. exhibited Ki values within an order of magnitude of the octopamine Ki) (Table 2).

**Table 2:** **Binding and larvicide activity of ZINC compounds**

ZINC COMPOUND	Ki (μM)	EC _50_ (μM)	IC _50_ (μM)	Larval bioassay (% mortality)
OA	7.7	0.017	-	nd*
10883478	34.2	-	55.3	74
02643656	14.9	5.6	-	nd
40118665	56.7	-	17.6	nd
48353678	2.7	22.0	-	nd
48345912	6	-	4.0	nd

*Not detectable.

To assess insecticidal activity, all 70 compounds in an *Ae aegypti* larvicide bioassay were tested.

Only 4 compounds induced any mortality. In the initial assay (19922690, 12434335, 28695014) induced a small amount (14-21%) of mortality compared to 0% in the DMSO alone group at 24 hours and so were not tested further. Compound 10883478, however, induced substantial mortality, killing 70.5 ± 20.5% of the larvae at 24 hours.

## Discussion

Octopamine is the most abundant biogenic amine in invertebrates and is virtually absent in vertebrates, making this pathway an attractive target for insecticide development with potential low toxicity in vertebrates. Often thought of as the functional equivalent of vertebrate norepinephrine, octopamine is an invertebrate neuromodulator, neurohormone and neurotransmitter. The octopaminergic system is involved in a variety of insect physiological processes, including locomotion, memory, mating, and egg laying [47]. Interestingly, cocaine, a natural insecticide, is believed to potentiate octopaminergic neurotransmission [48]. In addition, the formamidine pesticides, chlordimeform and amitraz are thought to act as octopamine agonists [49]. Here, two *An. gambiae* octopamine receptors, AgOAR45A and AgOAR45B were functionally characterized. Through *in silico* screening approaches one scaffold that holds promise for the development of a novel insecticide was identified.

We identified six full-length OAR/TyrR genes in the *An. gambiae* genome. Phylogenetic analysis revealed two β_2_-adrenergic like receptors, two TyrR, one OA/Tyr receptor and a gene that encodes two alternatively spliced OAR transcripts, *AgOAR45A* and *AgOAR45B*. All seven predicted transcripts shared the characteristic sevem TM domains characteristic of GPCRs. *AgOAR45A* and *AgOAR45B* are splice variants sharing the first three exons and differing only with the fourth exon. Both predicted proteins possess the conserved aspartic acid in TM3 and two of three conserved serines known to mediate binding of GPCRs to biogenic amines [50]. While identical for the first 246 amino acids, *AgOAR45A* and *AgOAR45B* only share 32% identity (46% similarity) at the C-terminal end of the protein. These amino acids encode the third intracellular loop and the C-terminus of the receptor thought to mediate the interaction between the GPCR and G-proteins [51, 52], invoking the possibility that the two splice variants couple to different G-Protein signaling pathways. However, these results revealed that AgOAR45A and AgOAR45B, both signal through Ca^++^ and cAMP, similar to their homologous proteins, DmOamb-B and DmOamb-D, in *Drosophila* [16, 53].

GPCRs generally have three closely grouped serine residues found in TM5 that are located in the binding site. AgOAR45B, however, retains only two of these serine residues. The results demonstrated that D100, S206 and S210 are important in the agonistic function of AgOAR45B to octopamine, similar to what has been demonstrated for other GPCR-Ligand combinations [9–11]. It should be noted however, that while the serine residues do appear to be important, the function of the protein is not solely dependent on these residues. Even with both serine residues replaced, AgOAR45B can still respond to higher concentrations of octopamine (Figure 5B). This observation suggests that another mechanism may be involved in the activation of this protein. One hypothesis is that the ligand may also be involved in π-π interactions (i.e. the attractive, non-covalent interactions that occur between aromatic rings with some of the aromatic residues) in the TM6 region (Figure 4). It seems possible that as the TM6 region moves towards the TM3 region in order to form these interactions, that the cytoplasmic end of the TM6 region moves outward. This hypothesis is supported by previous work in which the cytoplasmic end of TM6 region is shown to move outward when the protein is activated [27, 28].

One interesting scaffold group found to be effective contained mostly antagonists (48345912, 48354000 and 40118665) and one agonist (48353678). It is likely that this difference is due primarily to the length of the short chain to the pyridine group of 48353678, as compared to the longer chain to the indoline group of 48353672. It is possible that the shorter chain of 48353678 causes the active site to be more condensed and be more like the active conformation, while the longer chain of 48353672 allows the active site to assume a more open and inactive conformation. Docking of these compounds shows that both ligands can bind to the same residues in a similar conformation, yet the longer chain of 48353672 appears to be forcibly constrained and would preferably be extended, allowing the pocket to open.

Two of the common scaffolds that found docking to both agonist-bound and antagonist- bound AgOAR45B conformations, i.e., protonated piperazine and protonated -NR_2_, were also found in another study performed with the dopamine D3 receptor [54]. However, none of the active compounds identified contain protonated piperazine and only two contain protonated -NR_2_ (see Additional files 10 and 11). This, along with the presence of another scaffold that was not reported in the dopamine D3 receptor study, i.e., protonated imidazole (which 10883478 contains), indicates that there may be specificity for AgOAR45B to certain compound types.

Four of the compounds exhibited activity against mosquito larvae in an initial assay. Interestingly, these compounds were among the best antagonists and exhibited between 14 and 70.5% in a mosquito larval bioassay. Three (10883478, 12434335 and 28695014) of the four were imidazole derivatives with different carbon and nitrogen substitution patterns. Interestingly, a compound from the same scaffold as 10883478 that also exhibited potent antagonist activity was not active in the larvicide bioassay. Possibly, the ortho methyl substitution of 10883478 makes this compound less susceptible to metabolism.

## Conclusions

Here, two *An. gambiae* OARs were functionally characterized and a computational approach coupled with experimental methods was utilized to discover possible lead compounds for the development of novel insecticides. We identified two AgOAR45B agonists and twenty-one antagonists, with one antagonist exhibiting substantial larvicide activity in a mosquito bioassay. However, we were unable to directly attribute OAR antagonism to the killing activity because of potential off target effects at the high concentrations utilized. Further studies on all the compounds to enhance potency, increase stability, and assess possible off target effects are needed. In addition, refinement of the computational models utilized for virtually screening will increase the possibility of identifying additional compounds for development. Running longer conventional MD simulations for ensuring optimal states is computationally impractical; however, accelerated molecular dynamics (aMD) will allow simulations on the millisecond timescale in only hundreds of nanoseconds [48]. In addition, a solved crystal structure of AgOAR45B would facilitate a more accurate model of the protein for the *in silico* discovery of lead compounds.

## Electronic supplementary material

Additional file 1: **Primer sequences for mutagenesis of** ***AgOAR45B.*** (DOCX 15 KB)

Additional file 2: **Diagram of the basic computational-experimental workflow.** (ZIP 856 KB)

Additional file 3: **Alignment of AgOAR45B (AGAP000045B) with human b2-adrenergic receptor (NP_000015).** Black boxes indicate active site residues. AgOAR45B exhibits a 67% sequence active site identity (73% similarity) to the β_2_-adrenergic receptor active site. (ZIP 649 KB)

Additional file 4: **Genomic organization of** ***AgOAR45A and*** ***AgOAR45B.*** AGAP000045 region downloaded from VectorBase. Maroon boxes indicate protein-coding regions. B) Protein alignment of the two proteins performed using Geneious software. Red boxes indicate transmembrane regions. Note low identity after position 250. C) Hydropathy Plot for AGAP000045B. (ZIP 882 KB)

Additional file 5: **AgOAR45A and AgOAR45B Gαq signaling.** Dose response curves of AgOAR45A (circles) and AgOAR45B (squares) to known different concentrations of octopamine. Data are expressed as the change in fluorescence intensity (F_max_-F_min_) divided by the background fluorescence intensity (F_min_) % 1 μM octopamine response. Means ± SD of three independent experiments are presented. (ZIP 278 KB)

Additional file 6: **AgOAR45B homology models obtained from I-TASSER.** Blue indicates the inactive conformation while orange indicates the active conformation. Promethazine, shown in green, indicates the active site. Both sides of the proteins are presented, with (A) showing TM 1–5 and (B) showing TM 5–7, 2, and 1. Total Protein RMSD: 10 Å. TM-region RMSD: 2 Å. (ZIP 1 MB)

Additional file 7: **RMSDs of MD simulation trajectories compared to their respective initial conformations.** (A) RMSD MD simulation yielding antagonist-bound conformation presented in Figure 4A, max flux = 0.82Å. (B) RMSD MD simulation yielding antagonist-bound conformation presented in Figure 4B, max flux = 0.86 Å. (C) RMSD MD simulation yielding agonist-bound conformation presented in Figure 4C, max flux = 0.38Å. (D) RMSD MD simulation yielding agonist-bound conformation presented in Figure 4D, max flux = 0.47Å. (E) RMSD MD simulation yielding agonist-bound conformation presented in Figure 4E, max flux = 0.72 Å. Max flux values reported are the largest RMSD differences in the 10–20 ns simulation trajectories for the respective runs. (ZIP 328 KB)

Additional file 8: **Scaffolds obtained from antagonist-bound screens.** The most commonly observed interactions with D100 in the virtual screen were (A) protonated piperazine, (B) protonated imidazole and, (C) protonated -NR2. Figure (D) shows one of the other potential interactions that were observed. (ZIP 4 MB)

Additional file 9: **Scaffolds obtained from agonist-bound screens.** The most commonly seen interactions with D100 in the virtual screen were (A) protonated piperazine, (B) protonated -NR_2_/-NR_3_ and, (C) -NH-R-NH- (where D100 interacts with both nitrogens). Figure (D) shows one of the other potential interactions that were observed. (ZIP 6 MB)

Additional file 10: **Activity of Zinc compounds tested** ***in vitro.*** (DOCX 136 KB)

Additional file 11: **Zinc chemistries purchased and tested in the AgOAR45B reporter assay.** (DOCX 136 KB)

## Abbreviations

OAR: Octopamine receptor
GPCR: G-protein coupled receptor
MD: Molecular dynamics
TM: Transmembrane
qPCR: Quantitative PCR
RMSD: Root-mean-square deviation
POPC: 1- palmitoyl-2-oleoylphosphatidylcholine
ns: Nanoseconds
ps: Picoseconds
fs: Femtoseconds.
