# Supplementary material for: Characterization of the Anopheles gambiae octopamine receptor and discovery of potential agonists and antagonists using a combined computational-experimental approach
Source: Malar J. 2014 Nov 18;13:434. doi: 10.1186/1475-2875-13-434 (PMC4253978; doi:10.1186/1475-2875-13-434)
Supplement: Supplementary file 1 — Additional file 1: Primer sequences for mutagenesis of AgOAR45B. (DOCX 15 KB) [file 12936_2014_3608_MOESM1_ESM.docx]

| **Supplemental Table 1: Primer sequences for mutagenesis of *AgOAR45B*** | | | |
| --- | --- | --- | --- |
| Amino Acid | Substitution | Region | Oligonucleotide |
| D 100 | A | TM III | GGC TGG CGG TC**G** CCG TCT GGA TGT G |
| D 100 | N | TM III | CTG GCT GGC GGT CA**A** CGT CTG GAT GTG |
| E 161 | A | ECL II | CTG GTC GGG TGG AAG G**C**A CAG AA**A** GTG AAA GAG |
| E 161 | Q | ECL II | CTG GTC GGG TGG AAG **C**AA CAG AAG GTG AAA G |
| S 206 | A | TM V | GTA CGT CGT CTA C**G**C AGC CCT CGG CTC |
| S 210 | A | TM V | ACT CAG CCC TCG GC**G** CCT TCT ACA TTC CC |
