# Supplementary material for: Characterization of the Anopheles gambiae octopamine receptor and discovery of potential agonists and antagonists using a combined computational-experimental approach
Source: Malar J. 2014 Nov 18;13:434. doi: 10.1186/1475-2875-13-434 (PMC4253978; doi:10.1186/1475-2875-13-434)
Supplement: Supplementary file 11 — Additional file 11: Zinc chemistries purchased and tested in the AgOAR45B reporter assay. (DOCX 136 KB) [file 12936_2014_3608_MOESM11_ESM.docx]

| **Additional File 11: Zinc Chemistries purchased and tested in the AgOAR45B reporter assay.**  Compounds are sorted by Zinc ID. | | | | | |  |
| --- | --- | --- | --- | --- | --- | --- |
| Virtual  BINDING SCAFFOLD  (Antagonist or Agonist) Confirmation) | ZINC ID | Structure | Ligand Type | 100μM^a^  Antagonist Efficiency  (μM IC_50_) | 100μM^a^  Agonist Efficiency  (μM EC_50_) |  |
| Misc  (Antagonist) | 1757652 | 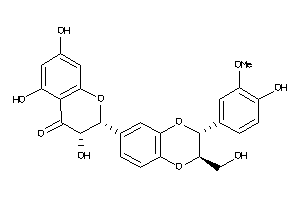 | not active | >90 | <5 |  |
| 2  (Antagonist) | 6283070 | 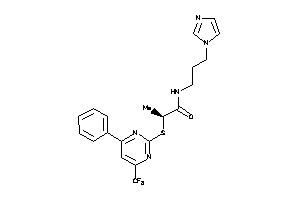 | not active | >90 | <5 |  |
| 9274026-like | 6791891 | 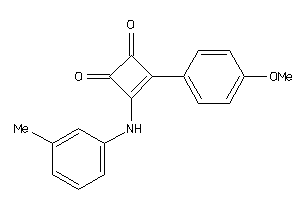 | weak  antagonist | 89.8^b^ | <5 |  |
| 10883478-like | 7205872 | 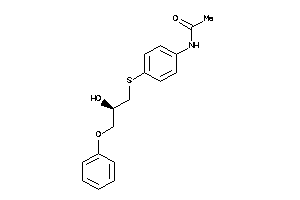 | not active | >90 | <5 |  |
| 2  (Antagonist) | 8694341 | 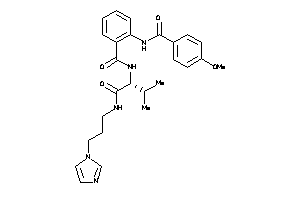 | not active | >90 | <5 |  |
| 9274026-like | 9273955 | 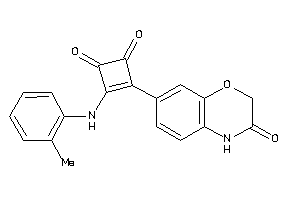 | weak antagonist | 70.1^b^ | <5 |  |
| Misc  (Antagonist) | 9274026 | 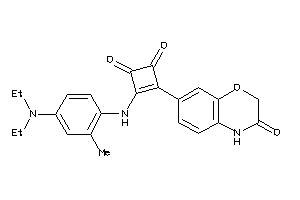 | antagonist | 11^b^  (13.2) | <5 |  |
| 9274026-like | 9274053 | 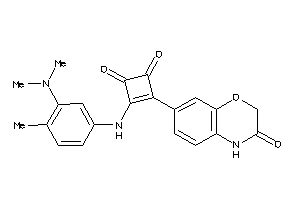 | antagonist | 55.9^b^ | <5 |  |
| 27417161-like | 00200688 | 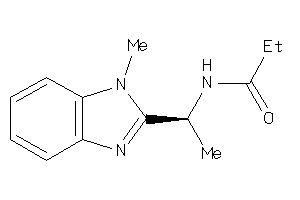 | antagonist | 74.2^b^ | <5 |  |
| 2  (Agonist) | 00441586 | 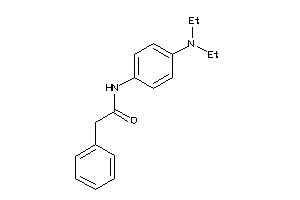 | weak agonist | 86.2 | 13  (3.7) |  |
| 2  (Agonist) | 02643656 | 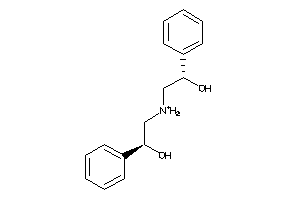 | agonist | >90 | 88.3  (5.6) |  |
| 27417161-like | 02721570 | 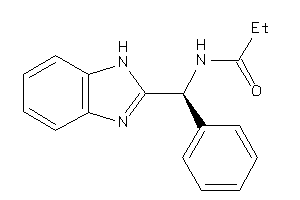 | not active | >90 | <5 |  |
| Misc  (Agonist) | 10883478 | 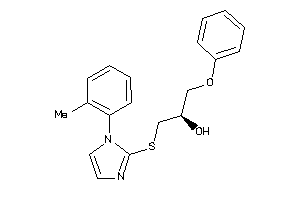 | antagonist | 17  (55.3) | <5 |  |
| 10883478-like | 11207230 | 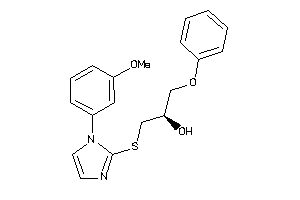 | not active | >90 | <5 |  |
| 2  (Antagonist) | 12434335 | 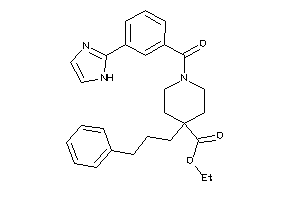 | antagonist | 21.8^c^ | <5 |  |
| 2  (Antagonist) | 12445779 | 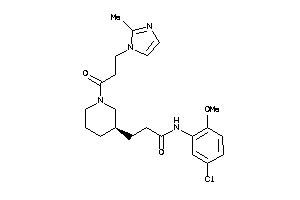 | weak antagonist | 74.6 | <5 |  |
| 27417161-like | 12484738 | 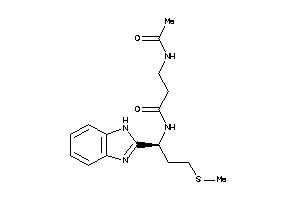 | antagonist | 63.8^b^ | <5 |  |
| 27417161-like | 12535485 | 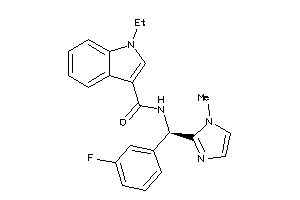 | weak antagonist | 92 | <5 |  |
| 10883478-like | 12547680 | 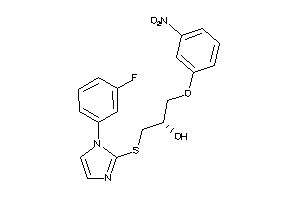 | antagonist | 11.6 | <5 |  |
| 2  (Antagonist) | 12790591 | 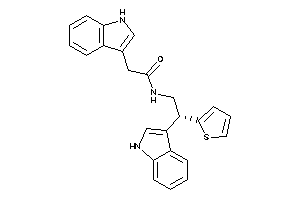 | antagonist | 10.1^c^ | <5 |  |
| 10883478-like | 12794746 | 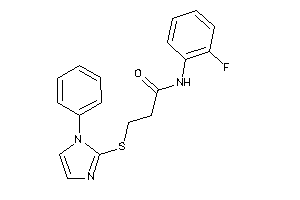 | not active | >90 | <5 |  |
| 2  (Agonist) | 12984232 | 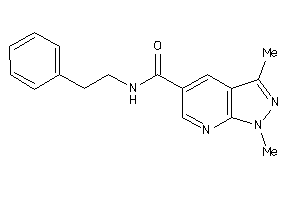 | weak  agonist | 142.5 | 6.1 |  |
| 2  (Antagonist) | 15671138 | 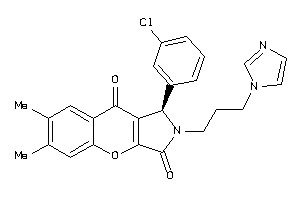 | antagonist | 22.6^c^ | <5 |  |
| 2  (Antagonist) | 15671140 | 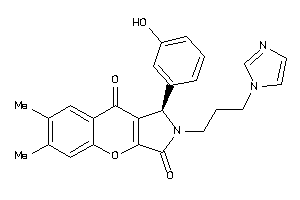 | not active | >90 | <5 |  |
| 9274026-like | 15725975 | 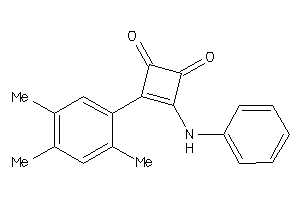 | antagonist | 44.8 | <5 |  |
| 2  (Agonist) | 19228760 | 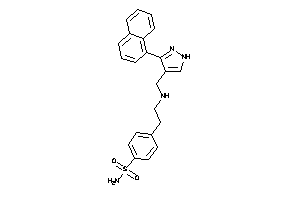 | not active | >90 | <5 |  |
| 2  (Antagonist) | 19635280 | 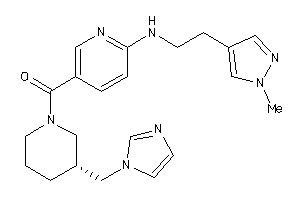 | not active | >90 | <5 |  |
| 2  (Agonist) | 19922690 | 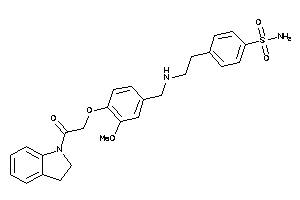 | antagonist | 1.7  (13.3) | <5 |  |
| 3  (Antagonist) | 20507208 | 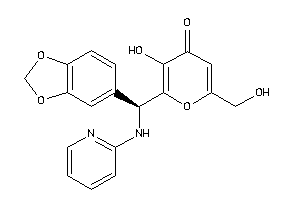 | not active | >90 | <5 |  |
| 2  (Agonist) | 20601736 | 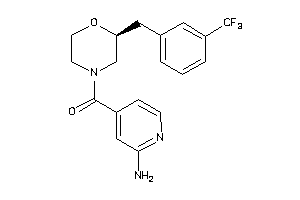 | not active | >90 | <5 |  |
| Misc  (Antagonist) | 21866120 | 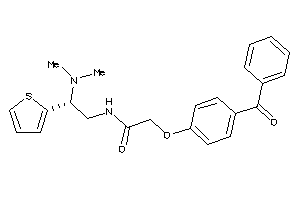 | weak antagonist | 68.3 | <5 |  |
| Misc  (Antagonist) | 22289086 | 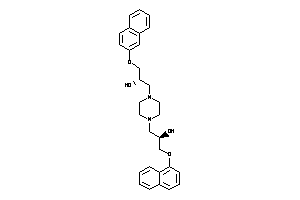 | not active | >90 | <5 |  |
| 2  (Antagonist) | 24023023 | 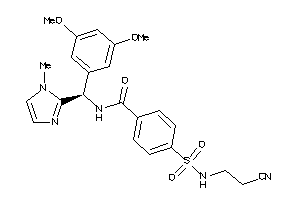 | not active | >90 | <5 |  |
| Misc  (Antagonist) | 24757679 | 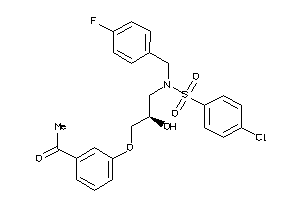 | antagonist | 47.6^c^ | <5 |  |
| 2  (Agonist) | 25175360 | 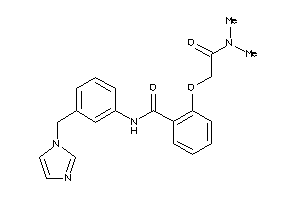 | not active | >90 | <5 |  |
| 2  (Antagonist) | 27417161 | 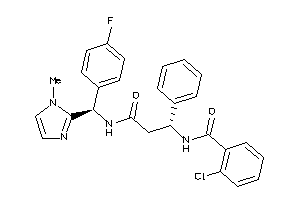 | weak  antagonist | 57.2^b^ | <5 |  |
| 2  (Agonist) | 28695014 | 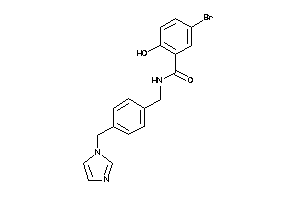 | antagonist | 34.9 | <5 |  |
| Misc  (Agonist) | 32322962 | 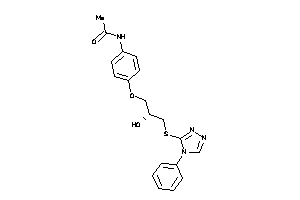 | weak antagonist | 89.2 | <5 |  |
| 27417161-like | 32843433 | 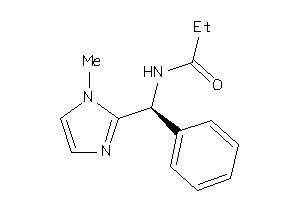 | weak antagonist | 89 | <5 |  |
| 2  (Antagonist) | 32860047 | 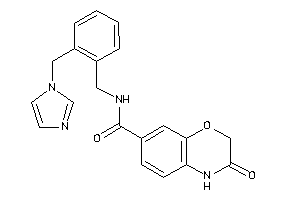 | weak antagonist | 55.8 | <5 |  |
| 2  (Agonist) | 32913027 | 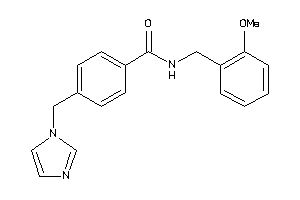 | not active | >90 | <5 |  |
| 2  (Agonist) | 33026561 | 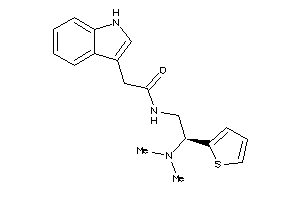 | not active | >90 | <5 |  |
| 1  (Agonist) | 33313914 | 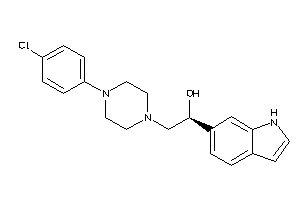 | weak agonist | >90 | 4.2  (4.4) |  |
| 2  (Agonist) | 34886371 | 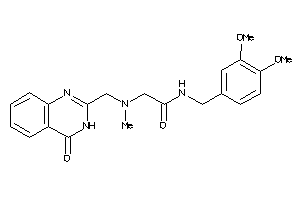 | not active | >90 | <5 |  |
| 10883478-like | 39985747 | 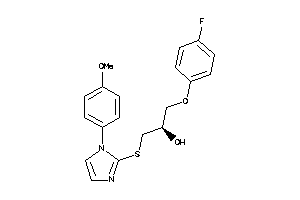 | weak antagonist | 79.5 | <5 |  |
| 48345912-like | 40118665 | 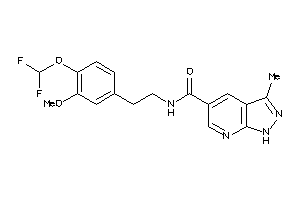 | antagonist | 13.4^b^  (17.6) | <5 |  |
| 2  (Antagonist) | 4148879 | 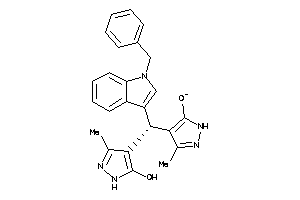 | antagonist | 35.9^c^ | <5 |  |
| Misc  (Antagonist) | 42437778 | 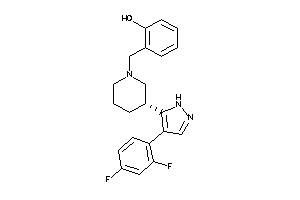 | antagonist | 3.4^c^ | <5 |  |
| 2  (Agonist) | 45606454 | 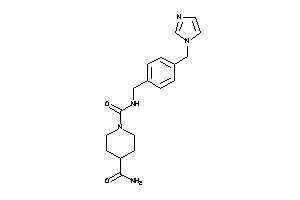 | not active | >90 | <5 |  |
| 1  (Antagonist) | 45966662 | 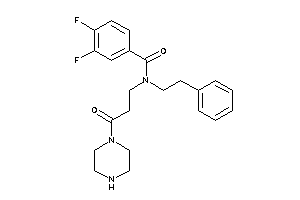 | not active | >90 | <5 |  |
| 1  (Antagonist) | 45966686 | 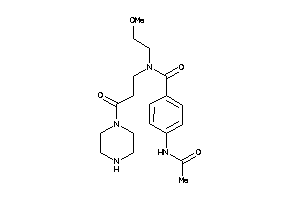 | not active | >90 | <5 |  |
| 1  (Antagonist) | 46140767 | 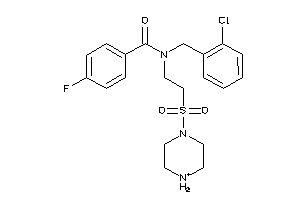 | antagonist | 35.1^c^ | <5 |  |
| 1  (Antagonist) | 46140913 | 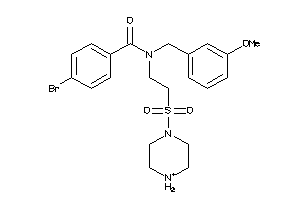 | antagonist | 34.4^c^ | <5 |  |
| 2 and 3  (Agonist) | 47579217 | 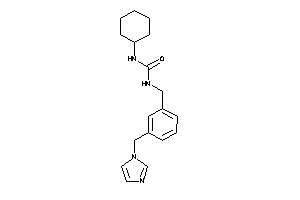 | not active | >90 | <5 |  |
| 3  (Antagonist) | 48194443 | 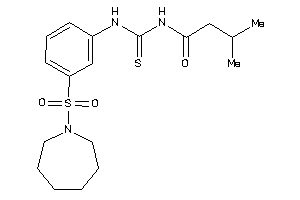 | weak agonist | >90 | 3  (27.9) |  |
| 2  (Agonist) | 48345912 | 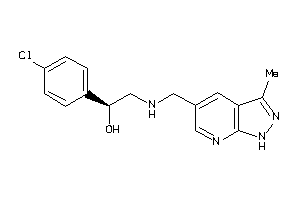 | antagonist | 16.9  (4.0) | 8.3 |  |
| 48345912-like | 48353672 | 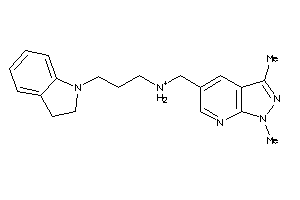 | antagonist | 35.4 | <5 |  |
| 48345912-like | 48353678 | 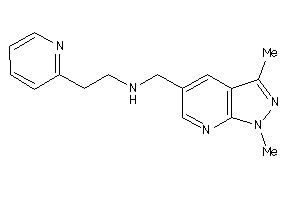 | agonist | >90 | 93.4  (22) |  |
| 48345912-like | 48354000 | 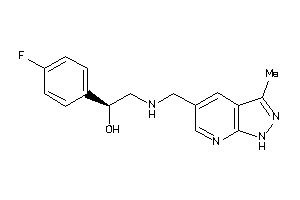 | antagonist | 30.9  (10.2) | 5.7 |  |
| 3  (Agonist) | 50063058 | 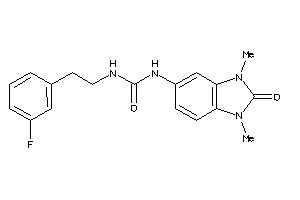 | weak antagonist | 53.9  (6.6) | <5 |  |
| 1  (Antagonist) | 55341583 | 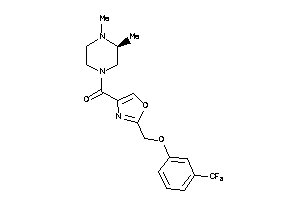 | weak antagonist | 89.6 | <5 |  |
| 2  (Agonist) | 55342789 | 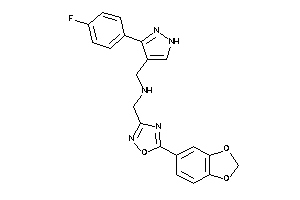 | weak antagonist | 88.6 | <5 |  |
| 10883478-like | 58007135 | 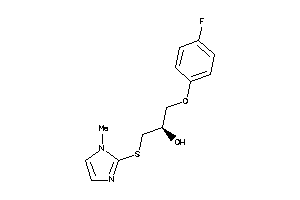 | not active | >90 | <5 |  |
| 10883478-like | 58171610 | 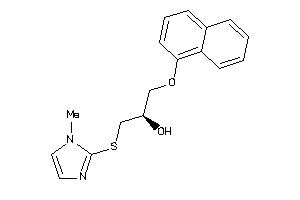 | weak antagonist | 83.9 | <5 |  |
| 2  (Agonist) | 58403478 | 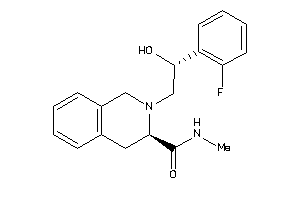 | weak  agonist | 89.5 | 6.1 |  |
| 10883478-like | 61715350 | 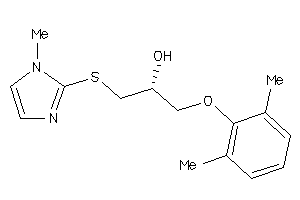 | not active | >90 | <5 |  |
| 10883478-like | 61715380 | 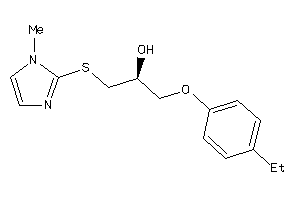 | not active | >90 | <5 |  |
| Misc  (Antagonist) | 65552607 | 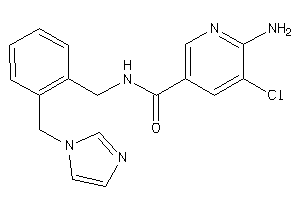 | weak antagonist | 67.3 | <5 |  |
| 2  (Agonist) | 69810774 | 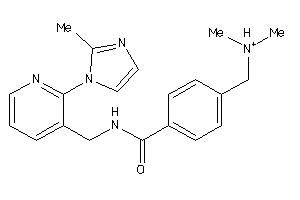 | weak antagonist | 65.3 | <5 |  |
| 2  (Antagonist) | 71799402 | 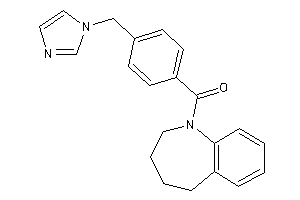 | weak antagonist | 72.2 | <5 |  |
| ^a^Percent of the 1μM octopamine response  ^b^Tested at 50μM because insoluble at 100μM  ^c^Compounds induced substantial cell death at 100μM and no activity at lower concentrations | | | | | | |
